# Estimating Measurement Error of the Patient Activation Measure for Respondents with Partially Missing Data

**DOI:** 10.1155/2015/270168

**Published:** 2015-11-17

**Authors:** Ariel Linden

**Affiliations:** ^1^Linden Consulting Group, LLC, Ann Arbor, MI 48103, USA; ^2^Department of Health Management & Policy, School of Public Health, University of Michigan, Ann Arbor, MI 48109, USA

## Abstract

The patient activation measure (PAM) is an increasingly popular instrument used as the basis for interventions to improve patient engagement and as an outcome measure to assess intervention effect. However, a PAM score may be calculated when there are missing responses, which could lead to substantial measurement error. In this paper, measurement error is systematically estimated across the full possible range of missing items (one to twelve), using simulation in which populated items were randomly replaced with missing data for each of 1,138 complete surveys obtained in a randomized controlled trial. The PAM score was then calculated, followed by comparisons of overall simulated average mean, minimum, and maximum PAM scores to the true PAM score in order to assess the absolute percentage error (APE) for each comparison. With only one missing item, the average APE was 2.5% comparing the true PAM score to the simulated minimum score and 4.3% compared to the simulated maximum score. APEs increased with additional missing items, such that surveys with 12 missing items had average APEs of 29.7% (minimum) and 44.4% (maximum). Several suggestions and alternative approaches are offered that could be pursued to improve measurement accuracy when responses are missing.

## 1. Introduction

Self-administered survey instruments are increasingly being used to identify individuals suitable for health management interventions [1]. Instrument validity typically depends on responses on all items. When items are missing, some instruments cannot be scored, while other instruments allow missing items but calculate the score based only on those items with responses (see McDowell [2] for a comprehensive guide to rating scales and questionnaires and their scoring algorithms). While the latter approach avoids the need to drop entire observations, missing items may limit the accuracy of the survey, leading to either under- or overestimation of the respondent's true score. Thus, depending on which survey instrument is implemented, program administrators are faced with the prospect of either losing entire observations or calculating scores that may be quite inaccurate, when any items on the survey are missing.

The patient activation measure (PAM) is a 13-question survey instrument that is gaining increased attention because of efforts to increase patient self-management with the goal of improving health outcomes and reducing cost [3]. The PAM assesses patient knowledge, skills, and confidence for self-management and has been demonstrated to be both valid and reliable for patients with and without chronic illnesses [4, 5]. Additionally, respondents can be stratified into 4 “stages of activation” according to their calculated PAM score, allowing for targeted interventions to support activation behaviors at different points along the continuum [5, 6].

The PAM may be scored when there are missing responses, which can lead to substantial measurement error. In turn, this error has serious implications for how the PAM is typically used in practice. First, when targeting individuals for an intervention to improve self-management skills, the goal is to identify individuals with low-activation and help move them up the activation scale, which should facilitate increasing knowledge and self-management skills. Individuals incorrectly categorized as “low-activation” (when they were in fact “high-activation”) would be invited to participate but would be unlikely to benefit. Conversely, patients incorrectly categorized as “high-activation” (when they were in fact “low-activation”) would be inappropriately excluded, leading to a missed opportunity to intervene. Second, when the PAM is used as an outcome measure for determining the effectiveness of an intervention, the measurement error introduced by scoring the PAM with missing items may bias the results. In this paper, we quantify the amount of error introduced by scoring the PAM across the full possible range of 1 to 12 simulated missing items.

The paper is organized as follows: In Section 2, the PAM instrument and the data set are briefly described; measurement error is defined, and the simulation methodology used to assess it is explained. In Section 3, the estimates of measurement error are presented by the number of missing items, both in the aggregate and separately by PAM level. In Section 4, the findings are summarized; and the implications for those using the PAM for engaging individuals in health management interventions and for evaluations using PAM as an outcome measure are discussed. Finally, existing methods that are more suitable for dealing with incomplete survey data are briefly described.

## 2. Methods

### 2.1. Patient Activation Measure

The PAM is comprised of 13 questions about beliefs, confidence in managing health related tasks, and self-assessed knowledge, with each response representing the degree of agreement or disagreement with each statement. Based on the responses to the 13 items, each individual is assigned an activation score ranging from 0 to 100 [3].

For patient engagement purposes, the PAM is typically stratified into 4 “stages of activation” according to the calculated score [5, 6]. Individuals categorized as level 1 typically do not yet believe that they must play an active role in their own health; they may feel that they should be a passive recipient of care. Individuals categorized as level 2 may lack the basic facts or not have connected basic facts to a broader understanding about their health or recommended health behaviors. Individuals categorized as level 3 have key facts and are beginning to take action but may lack the confidence and skill to support their behaviors. Individuals categorized as level 4 have adopted new behaviors but may not be able to maintain them when faced with stressors or health crises [6].

### 2.2. Data

Data in this study come from a parallel-group, stratified, randomized controlled trial, in which 512 patients hospitalized at two community hospitals, with congestive heart failure (CHF) or chronic obstructive pulmonary disease (COPD), were randomly assigned to the intervention (*n* = 253) or usual care (*n* = 259). The intervention encompassed a 90-day hospital-based transitional care program. The primary endpoints were 30- and 90-day all-cause readmissions. Secondary measures included all-cause emergency department (ED) visits and mortality (see Linden and Butterworth [7] for details).

To assist in providing a targeted intervention, participants completed the PAM survey at baseline (during enrollment), 30 days, and 90 days. All 512 participants completed the survey at baseline, 386 completed the survey at 30 days (75.4%), and 372 completed the survey at 90 days (72.7%). In examining the surveys with missing data, no distinct patterns of missing values were detected.

For the present study, 132 of the original surveys were excluded if there were missing item scores among one or more of the 13 questions or if a participant's total PAM score was a perfect 100 (which we would consider implausible in a sick population). Thus, 1,138 “clean” surveys were retained for the current analysis and serve as the unit of measure.

### 2.3. Analytic Approach

The analytic process was comprised of four steps: (1) 1,000 simulations of a missing-data generating process, (2) calculation of PAM scores for each simulation, (3) calculation of summary statistics for each of the 1,138 surveys used in the simulations, and (4) calculation of summary statistics across all simulations for all surveys.

In the first step, to simulate one missing value, a random number between 1 and 13 was generated for each of the 1,138 existing surveys in the data. The resulting value was then cross-referenced to the corresponding question number, and the actual item response was replaced with a missing value. For example, if, for a given survey, the random number generated was 4, then the actual item response in question 4 was replaced with a missing value. This process was repeated 1,000 times. To simulate a greater number of missing values (i.e., from two to twelve), an identical process was followed. For example, to simulate six missing values, six random numbers between 1 and 13 were generated, and the corresponding questions were replaced with missing values. Each survey therefore had 12,000 corresponding simulated surveys containing randomly missing items, starting at 1,000 iterations with one missing item, and ending with 1,000 iterations for 12 missing items. This process generated a total of 13,656,000 simulated surveys.

In the second step of the analytic process, a PAM score was calculated for each of the simulated surveys using the proprietary PAM algorithm (http://www.insigniahealth.com/).

In the third step, summary statistics were calculated within the simulation results for each of the 1,138 surveys for each level of missing responses (1–12). Specifically, the mean simulated PAM score was computed by calculating the mean of all 1,000 simulated PAM scores at a given level of missing responses. Likewise, the minimum and maximum simulated PAM scores were located among the 1,000 simulated PAM scores. The absolute percentage error (APE) was utilized as a generalizable measure of accuracy [8, 9]. This measure represents the absolute error between the true mean and the simulated mean, minimum, and maximum values derived from simulation and is calculated as the absolute value of the following: (true mean − simulated mean)/true mean *∗* 100 (where the simulated mean is substituted with the simulated average minimum and maximum for those metrics, accordingly).

For example, assume that a given survey has a true PAM score of 70.0 (when scored with no missing items) and we run 1,000 simulations for the missing response level of three (i.e., 3 randomly chosen items are replaced with missing values). The simulated mean represents the average of these 1,000 simulated PAM scores, which we will assume as 65.0. Assume further that, among the 1,000 simulated PAM scores with three missing items, the lowest score (min) was 47.4 and the highest score (max) was 82.8. Therefore, for this survey with three missing items, APE (mean) = abs((70.0 − 65.0)/70.0)*∗*100 = 7.1%; APE (min) = abs((70.0 − 47.4)/70.0)*∗*100 = 32.3%; APE (max) = abs((70.0 − 82.8)/70.0)*∗*100 = 18.3%. Taken together, these statistics represent how different the true PAM score is from the mean, lowest, and highest simulated PAM scores. The min and max values, in particular, carry important meaning, as they represent the extreme bounds of how inaccurate the extrapolated scores may be.

In the fourth step, the summary statistics calculated at the individual-level were aggregated across all the simulated surveys, within a given missing response level and by the four PAM levels [5, 6]. Specifically, the overall simulated average PAM score was computed by taking the average of all individual-level simulated mean PAM scores, and the overall simulated average minimum and maximum PAM scores were computed by taking the average of all individual-level simulated minimum and maximum PAM scores. Additionally, computing these statistics by PAM level allows for an assessment of the extent to which measurement error creates overlap across levels.

## 3. Results

Table 1 presents the characteristics of the study population (*N* = 512). In general, participants were predominately female, married (or living with a caregiver), over age of 65, insured by Medicare, and sick with several comorbidities. Participants had also utilized substantial acute hospital services in the prior year.

Across the 1,138 complete surveys used in the current study, PAM scores ranged from 8.2 to 91.6, with a mean of 57.2 (s.d. = 13.5) and a median of 56.4 (IQR = 21.1). This wide distribution of scores suggests that the results of the simulations in the current study should generalize to most other populations [10].

**Figure 1:**
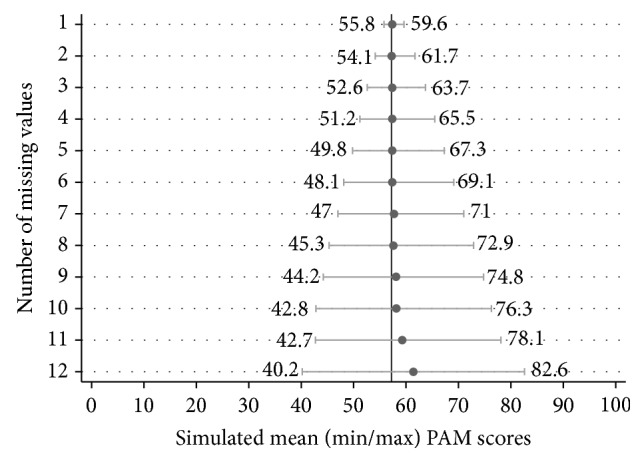
Mean, minimum, and maximum patient activation measure (PAM) scores derived via simulation (1,000 repetitions for each missing value level) on the entire study population. The vertical line represents the true mean of all PAM scores = 57.2.

**Figure 2:**
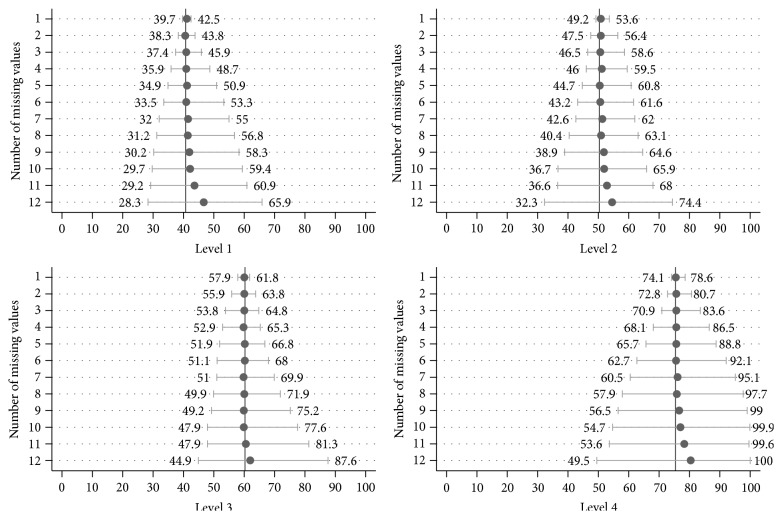
Mean, minimum, and maximum patient activation measure (PAM) scores derived via simulation (1,000 repetitions at each missing value level), by PAM level. Vertical lines represent the true mean of all PAM scores per given level (40.7, 50.3, 60.3, and 75.4, for levels 1 through 4, resp.).

Figure 1 depicts the average simulated mean, minimum, and maximum PAM scores for each number of missing item responses for the overall study population and Table 2 provides the corresponding average APE for each of these measures. The vertical line in Figure 2 represents the true mean PAM score of 57.2; the filled circles represent the simulated means; and the error bars represent the simulated average minimum and maximum scores. Both visually and numerically, we find that the simulated mean begins to differ from the true mean at about 9 missing item responses, where the APE of the mean doubles from 0.7% to 1.5%. This difference increases as the number of missing item responses increases. With 12 missing responses, the simulated mean is a full 4 points higher than the true mean (61.2 versus 57.2), which results in an average APE of 7.3%.

Second, and more importantly, there is a close to monotonic increase in the minimum and maximum range of scores by the number of missing item responses. For example, with one missing response, the simulated mean PAM score was 57.4, the simulated average minimum score was 55.8, and the simulated average maximum score was 59.6. Thus the minimum PAM score was, on average, 1.6 points below the mean simulated PAM score and the maximum PAM score was, on average, 2.2 points above the mean simulated PAM score (with corresponding average APEs of 2.5% and 4.3%). With two missing responses, the mean simulated PAM was unchanged, but the minimum PAM was, on average, 3.2 points below the mean simulated PAM and the maximum PAM was, on average, 4.4 points above the simulated mean (with corresponding APEs of 5.4% and 7.8%). At the point in which 12 missing responses were generated, the average minimum PAM was a full 21.1 points below the simulated mean PAM, and average maximum PAM was 21.3 points above the simulated mean PAM score (with corresponding APEs of 29.7% and 44.4%).

Figure 2 illustrates the simulated mean, minimum, and maximum PAM scores for each number of missing items, according to each of the four PAM levels [5, 6]. As shown, the general patterns across the four PAM levels were consistent with that of the aggregated PAM scores. That is, there is a departure between the simulated mean PAM score and the true mean PAM score at about 9 missing items (with the simulated mean always being higher than the true mean) and a consistent increase in the average minimum and maximum range of scores with each additional missing item.

## 4. Discussion

The results of this simulation analysis indicate that calculating PAM scores when survey item responses are missing may result in substantial measurement error. With only one missing item, the calculated score may be, on average, either 2.5% lower or 4.3% higher than the true score. This error increases monotonically with additional missing responses, so that a survey with 12 missing items may be scored, on average, as low as 30% lower than the true score or as high as 44% higher than the true score.

There are two important implications of these findings. First, when the PAM is used as a guide for triaging individuals to a specific level or type of an intervention, PAM scores calculated with missing items must be considered with caution. Depending on the number of missing items, the calculated score may possibly reclassify an individual as far as three levels higher (or lower) than the level based on the “true” (no missing items) PAM score. Given these findings, interventions should be cautious when using the PAM to identify individuals for intervention based on surveys with any missing items. To ensure no misclassification of patients to intervention groups based on their PAM level, the only robust approach is to simply not score any survey with missing items.

The second implication of these results is that when the PAM is used as an outcome measure for determining the effectiveness of an intervention, the measurement error introduced by scoring the PAM with missing items may bias the results. This suggests that, at the very least, investigators should provide details of the pattern of item nonresponse when presenting the results of their research. Readers will thus be better able to assess the quality of the reported outcomes. This also suggests that evaluators consider using multiple imputation [11] or other statistical techniques for handling missing item scores in the PAM, rather than the recommended practice of extrapolation. In general, multiple imputation estimates the missing items *M* times according to a statistical model. The resulting *M* different complete data sets are then analyzed using standard statistical procedures. Multiple imputation corrects estimates and their standard errors for the uncertainty caused by the missing data [11]. For example, Liu et al. [12] used multiple imputation techniques for imputing SF-12 scores for respondents with missing data and found that the algorithms produced relatively accurate estimates of the true scores. As a sensitivity analysis [13], evaluators should consider first comparing treatment effect estimates for the subset of subjects with no missing responses and then again on the entire study population, using multiple imputed data. Such an approach was taken by Bordeleau et al. [14] to assess whether women with metastatic breast cancer participating in a group psychosocial intervention had higher Health related Quality of Life (HrQOL) scores than controls. In that study, all techniques provided similar results.

In summary, this study has several strengths, including the use of surveys collected under controlled conditions which likely result in more accurate responses, a wide range of survey scores which likely improve generalizability [10], and a large number of randomly generated simulations which likely replicate the full possible range of survey response (and nonresponse) patterns [15]. However, a key limitation is that the study population was older and sick with several comorbidities. While this may mean that the results do not generalize to a younger, healthier population, these individuals are not typically the focus of health management interventions.

## 5. Conclusion

The results of this analysis indicate that scoring PAM surveys with missing responses may lead to substantial measurement error, ranging from as low as 2.5% to as high as 44%, depending on the number of missing items. These results have important implications both for those using the PAM as a guide for assigning individuals to an intervention and for those who are using the PAM as an outcome measure in their research. The former can be addressed by simply not scoring any survey with missing items, and the latter can be addressed by either limiting the evaluation to only those surveys with nonmissing responses or using statistical approaches specifically designed to account for missing items. And while such models may require a statistician, the improvement in accuracy will likely translate into better information regarding the true effectiveness of interventions targeting patient activation as an outcome.

## Figures and Tables

**Table 1:** Baseline characteristics of all study participants [7].

Characteristic	Number (%)^*∗*^
Sample size	512
Primary condition	
CHF	257 (50.2%)
COPD	255 (49.8%)
Female	295 (57.6%)
Age, mean (SD)	66.75 (11.99)
Insurance	
Medicare	358 (69.9%)
Medicaid	52 (10.2%)
Commercial	69 (13.5%)
None	33 (6.4%)
Living conditions	
With spouse/caregiver	332 (64.8%)
Alone	169 (33.0%)
Other	6 (1.2%)
Homeless	5 (1.0%)
Comorbidities	
Cerebrovascular disease	224 (43.8%)
Diabetes	204 (39.8%)
Obesity	172 (33.6%)
Chronic pain	128 (25.0%)
Renal disease	117 (22.9%)
Acute myocardial infarction	103 (20.1%)
Peptic ulcer disease	87 (17.0%)
Depression	70 (13.7%)
Cancer	57 (11.1%)
Mental disorder	63 (12.3%)
Liver disease	40 (7.8%)
PAM, mean (SD)	54.03 (13.69)
Hospital utilization in prior 12 months	
Admissions (all-cause), mean (SD)	1.78 (1.32)
Hospital days (all-cause), mean (SD)	8.40 (8.65)
ED visits (all-cause), mean (SD)	0.91 (1.85)
LOS of index admission, mean (SD)	5.24 (4.05)

PAM: patient activation measure; ED: emergency department; LOS: length of stay.

^*∗*^Unless otherwise noted.

**Table 2:** Average absolute percent errors (APEs) between true PAM mean scores and simulated mean, minimum, and maximum scores, overall and separately by PAM levels.

Missing	Overall			Level 1			Level 2			Level 3			Level 4		
	APE (mean)	APE (min)	APE (max)	APE (mean)	APE (min)	APE (max)	APE (mean)	APE (min)	APE (max)	APE (mean)	APE (min)	APE (max)	APE (mean)	APE (min)	APE (max)
1	0.3%	2.5%	4.3%	0.9%	2.4%	4.4%	0.9%	2.2%	6.4%	0.5%	3.9%	2.6%	0.2%	1.6%	4.3%
2	0.2%	5.4%	7.8%	0.3%	6.0%	7.7%	1.0%	5.6%	12.0%	0.4%	7.3%	5.8%	0.4%	3.4%	7.1%
3	0.3%	8.0%	11.4%	0.6%	8.1%	12.9%	0.5%	7.6%	16.5%	0.2%	10.7%	7.5%	0.4%	5.9%	10.9%
4	0.3%	10.5%	14.4%	0.5%	11.9%	19.7%	1.6%	8.6%	18.3%	0.7%	12.1%	8.3%	0.4%	9.7%	14.8%
5	0.4%	13.0%	17.6%	1.1%	14.2%	25.1%	0.4%	11.1%	20.8%	0.1%	13.9%	10.8%	0.5%	12.9%	17.8%
6	0.3%	15.9%	20.9%	0.5%	17.7%	30.9%	0.5%	14.1%	22.3%	0.0%	15.2%	12.8%	0.3%	16.8%	22.1%
7	0.9%	17.8%	24.1%	2.0%	21.3%	35.2%	2.0%	15.4%	23.3%	0.7%	15.4%	16.0%	0.9%	19.7%	26.2%
8	0.7%	20.8%	27.4%	1.6%	23.4%	39.5%	1.2%	19.7%	25.5%	0.3%	17.3%	19.3%	0.6%	23.2%	29.6%
9	1.5%	22.7%	30.8%	3.2%	25.8%	43.2%	2.9%	22.8%	28.4%	0.7%	18.3%	24.8%	1.7%	25.0%	31.4%
10	1.8%	25.2%	33.3%	3.7%	26.9%	45.8%	3.2%	27.1%	31.0%	0.6%	20.6%	28.8%	2.1%	27.4%	32.6%
11	3.7%	25.4%	36.5%	7.1%	24.5%	49.6%	5.1%	27.3%	35.2%	0.4%	20.6%	34.9%	3.9%	28.9%	32.1%
12	7.3%	29.7%	44.4%	14.7%	30.3%	62.0%	8.4%	35.8%	47.9%	2.9%	25.5%	45.4%	6.7%	34.3%	32.7%
